# Slow oscillations promote long-range effective communication: The key for memory consolidation in a broken-down network

**DOI:** 10.1073/pnas.2122515119

**Published:** 2022-06-22

**Authors:** Hamid Niknazar, Paola Malerba, Sara C. Mednick

**Affiliations:** ^a^Department of Cognitive Sciences, University of California, Irvine, CA 92697;; ^b^The Ohio State University School of Medicine, Columbus, OH 43215;; ^c^Center for Biobehavioral Health, Research Institute at Nationwide Children's Hospital, Columbus, OH 43215

**Keywords:** effective connectivity, slow oscillations, memory consolidation, causal information flow, SO-spindle coupling

## Abstract

A prominent and robust finding in cognitive neuroscience is the strengthening of memories during nonrapid eye movement (NREM) sleep, with slow oscillations (SOs;<1Hz) playing a critical role in systems-level consolidation. However, NREM generally shows a breakdown in connectivity and reduction of synaptic plasticity with increasing depth: a brain state seemingly unfavorable to memory consolidation. Here, we present an approach to address this apparent paradox that leverages an event-related causality measure to estimate directional information flow during NREM in epochs with and without SOs. Our results confirm that NREM is generally a state of dampened neural communication but reveals that SOs provide two windows of enhanced large-scale communication before and after the SO trough. These peaks in communication are significantly higher when SOs are coupled with sleep spindles compared with uncoupled SOs. To probe the functional relevance of these SO-selective peaks of information flow, we tested the temporal and topographic conditions that predict overnight episodic memory improvement. Our results show that global, long-range communication during SOs promotes sleep-dependent systems consolidation of episodic memories. A significant correlation between peaks of information flow and memory improvement lends predictive validity to our measurements of effective connectivity. In other words, we were able to predict memory improvement based on independent electrophysiological observations during sleep. This work introduces a noninvasive approach to understanding information processing during sleep and provides a mechanism for how systems-level brain communication can occur during an otherwise low connectivity sleep state. In short, SOs are a gating mechanism for large-scale neural communication, a necessary substrate for systems consolidation and long-term memory formation.

## Significance

This work introduces a noninvasive approach to understanding information processing during sleep. Our results demonstrate that slow oscillations (SOs) provide the temporal-topographical event framework whereby long-range information flow increases dramatically compared to the relatively local activity patterns of the rest of sleep. The findings represent a conceptual leap in understanding how SOs unlock memory consolidation in a broken-down network, which is by promoting long-range, effective communication. This research will promote further investigations of understanding how brain oscillations alone and in nested rhythms promote network communication, as well as investigate how these properties vary and predict patterns of deficits in clinical populations and aging humans.

Human brain oscillations measured by electroencephalography (EEG) reflect the synchronized activity of thousands to millions of neurons, with spike timing modulated by the phase of ongoing oscillations (1). Current models propose that oscillations, separately or in coordination with one another, serve to organize information processing and communication in neuronal cortical networks in a state-dependent manner (2) and are thought to be markers of neural processing that occur in relation to specific behavior. A growing body of studies using neuronal recordings (3), cortical and local field potential (4), EEG signals, and cognitive and behavioral methods (5) have demonstrated the important and causal role of nonrapid eye movement (NREM) slow oscillations (SOs; <1 Hz) in brain function.

SOs are generally understood to be large traveling waves that coordinate activity globally across cortical regions and in cortical–subcortical interactions (6, 7) and have been shown to support memory formation (8, 9). According to the framework of systems consolidation, long-term memories (LTMs) are initially bound by a fast-learning system in the hippocampus (i.e., encoding), followed by stabilization of these memory traces in cortical stores during NREM (i.e., consolidation) (10). One potential way NREM may accomplish this process is by synchronizing neuronal activity along cortico-thalamo-hippocampal circuits via global SOs that coordinate nested spindles (sigma power; 12 to 15 Hz) and sharp wave ripples (11–14). Indeed, triple-phase coupling of these rhythms is associated with memory improvement, and causal interventions that block or enhance SO activity alter memory performance, suggesting a critical role of SOs in this process (15).

This prominent hypothesis of sleep-dependent memory, which posits increased activation of global communication networks, stands in opposition to the known properties of NREM sleep, which is characterized by reduced plasticity and a loss of network communication. Studies have shown that long-term potentiation, a form of synaptic plasticity that is the leading physiological model for the initial encoding and subsequent stabilization of memory, is actively suppressed during NREM sleep (16) compared with rapid eye movement (REM) sleep or an anesthetized state (17). At the systems level, using a combination of transcranial magnetic stimulation and high-density EEG, Massimini and colleagues (18) demonstrated a breakdown of transcallosal and long-range neural communication as the brain transitions from wake to the deeper stages of NREM sleep, suggesting that network communication during NREM sleep is highly local. Thus, the fundamental properties of NREM sleep (reduced synaptic plasticity; local, disconnected network) and the hypothesized mechanism of SO-driven systems consolidation (coordinated, global network activation) appear contradictory and cannot explain how NREM supports memory consolidation. From a theoretical perspective, the kind of memory consolidation we consider here rests upon long-term potentiation or depression, namely, activity-dependent plasticity. The requisite synaptic activity depends upon directed (effective) connectivity (i.e., ongoing communication in neuronal networks). Current formulations of this plasticity speak to long-term depression and synaptic regression. This view emerges from both empirical studies (19–21) and theoretical considerations based upon optimizing generative models (22, 23). In these accounts, emphasis is placed upon removing redundant or exuberant synaptic connections during sleep to ensure generalization (i.e., retaining the associations that matter).

The current study addresses the apparent paradox of NREM sleep supporting plastic activity-dependent changes in a globally coordinated network by investigating directional information flow during NREM sleep epochs with and without SOs. Prior studies in humans have typically assessed the functional role of sleep in memory at a distance, whereby memories are encoded, followed by a subsequent sleep period, after which memories are retrieved. This strategy does not allow for understanding of how the ongoing processing of sleep directly affects the consolidation of memories. To take a first step in amending this gap, we adopted an event-related approach to measure causal information flow across the brain during NREM sleep, specifically comparing different phases of the SO with the non-SO windows. In addition, studies typically analyze SOs using functional connectivity to measure temporal similarity or correlations between different EEG channels (24), which is limited to correlational measures and cannot identify directional causal communication. In contrast, we leveraged effective connectivity, which tests the influence that one neural system exerts over another either directly or indirectly (25), estimated using Granger causality. According to this approach, a causal relation is detected if past values of a primary signal (source) help predict a second signal (sink) beyond the information contained in its past alone (26). Thus, the current study noninvasively probes how the brain organizes network activity during sleep to support memory by measuring event-related causal information flow across EEG channels during the SO.

## Causal Information Outflow from Sources during SOs.

**Fig. 1.**
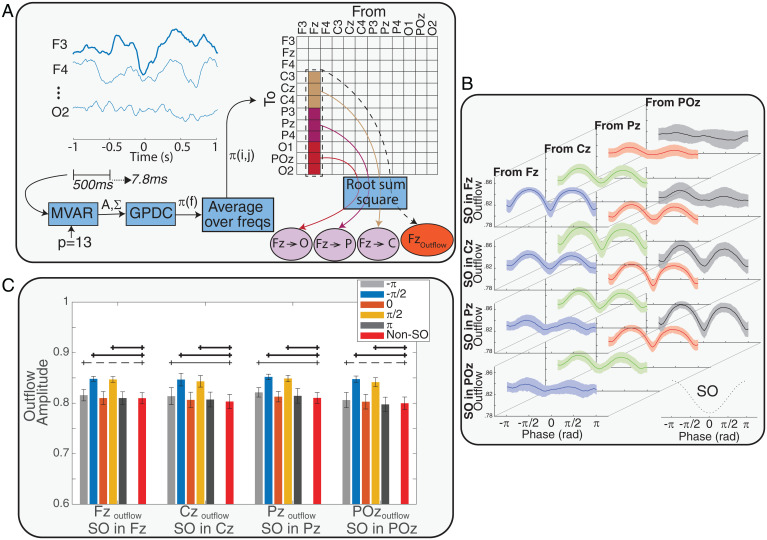
The process of causal information flow estimation over the phase of SOs and non-SO windows. (*A*) A representation of the process that estimates causal information flow between EEG channels. In summary, after SOs were detected, an MVAR model was fit on each 500-ms window of 12 EEG channels from 1 s before to 1 s after each SO trough, with stride of 7.8 ms. By using the GPDC method and averaging over all frequencies (freqs), the effective connectivity matrix (π) and the two defined quantifiers were calculated. After the quantifiers time series were resampled based on the SO phase, the phase series were averaged over all SOs of each subject to obtain the phase series of 1) the outflow from the sources (e.g., Fz_Outflow_) and 2) the flow from sources to sinks (e.g., Fz→Cz) for each subject. (*B*) Variation of outflow from all sources conditioned on the SO channel. Each row shows the outflow variation across the subjects over phase of SOs when the SO was detected in the represented channel. In each row, the columns show outflow variation in each of the sources (mean ± SD) over the phase of SOs in radian (rad). The phase series and their variation across the sources and SO channel show peaks before and after the SO trough and a possible relation between source and SO channel. The results in this figure suggest that sources closer to SO channels had greater height in outflow peaks. (*C*) Comparing outflows at five different phases of the SO (SO phase = {−π, π/2, 0,−π/2, π}) to outflow in non-SO windows. The error bars represent mean ± SD of outflow amplitude. ANOVA and post hoc tests found that peaks in −π/2 (prepeak) and π/2 (postpeak) phases had significantly larger outflow, while there were no significant differences between outflow in non-SO windows and the SO trough. Thick black lines indicate a strong significant difference (*P* value <0.005), thin lines indicate a significant difference (*P* value <0.05), and dashed lines indicate a marginal difference (0.05 < *P* value < 0.1) between SO and non-SO windows.

We used generalized partial directed coherence (GPDC) (27) as an estimator of causal information flow between different EEG channels. GPDC was defined as an extension of partial directed coherence (PDC) (28) to make it more robust against the amplitude scale of time series (see *Materials and Methods*, Effective Connectivity Estimation section for details). Four EEG channels (Fz, Cz, Pz, and POz) were considered as the source of information flow, and 12 channels (three channels in each frontal, central, parietal, and occipital regions) were considered as the sink of information flow. An automatic system detected SOs in the source channels, and two quantifiers were computed in the window 1 s before to 1 s after the SOs trough (Fig. 1*A*). We defined the first quantifier as the outflow from a source ($CH_{\mathrm{outflow}}$; “channel outflow”) and the second quantifier as flow from a source channel to a sink region (CH→R). Both quantifiers were estimated with respect to the SO phase and computed as an average for each subject (see *Materials and Methods* for definitions and details). Fig. 1*B* shows the average and SD of $CH_{\mathrm{outflow}}$ for each source across SO channels and based on SO phase. As SO phase changes, outflow peaks at −π/2 (a “prepeak”), drops to a local minimum at 0 (corresponding to the SO trough), and peaks again at π/2 (a “postpeak”). As a comparison, we also estimated outflow from sources during non-SO windows. We selected 500-ms-long time windows for all SOs in a randomized manner; values were chosen among the four source channels and in either sleep stage (Stage 2 and slow wave sleep [SWS]), with the only condition being that the time epochs had to be more than 10 s away from any SO trough. Fig. 1*C* displays the outflow values for each source in pre- and postpeaks, the SO trough, and non-SO windows. We tested outflow differences in SO and non-SO windows with ANOVA and post hoc analysis. The results showed that outflow ($CH_{\mathrm{outflow}}$) in the pre- and postpeaks was significantly larger than outflow in non-SO windows (*P* value <0.001), but outflows at SO troughs and the non-SO windows were not different (*P* value >0.05) (Fig. 1*C*). Also, compared to non-SO windows, there was no significant difference between outflow in half a cycle after SO trough (phase = π) and significant or marginally significant (0.05 < *P* value < 0.1) difference between outflow in − π phase, which can be caused by the effect of a prior SO on the successive SO in a sequence of SOs.

To investigate if high amplitudes during SOs were driving these peaks in information flow, we tested if there was a correlation between peaks of information outflow and amplitude of SOs in their relevant time point. The results showed that for each subject there were no significant relations between SO trough amplitudes in each channel and the outflow peaks from the relevant channels (*SI Appendix*, Fig. S1). Also, there were no significant linear relations between SO amplitude averages in each channel and the average of outflow peaks from the relevant channel (*SI Appendix*, Fig. S2).

We then asked if there was a structure underlying how high the peaks of information flow would be based on the various elements that allowed their estimate (locations of sources and sinks, etc.). To estimate the relative contribution to peak height of all these elements in an unbiased and comprehensive model, we fit a linear mixed-effects (LME) model to examine the effect of sources, SO channels, and distances between sources and SO channels ($D_{CH_{\mathrm{source}},CH_{SO}}$) and peak phase (i.e., whether the peak preceded or followed the SO trough) on the height of the peaks in information outflow. We assigned discrete values for the distances $D_{CH_{\mathrm{source}},CH_{SO}}$ between source (Fz, Cz, Pz, and POz) and SO channel as 0 to 3 in the linear model, adding 1 for each “step” in the frontal–occipital axis. We used a simple model to calculate the distances, as we considered distance between each two neighbor regions equal to 1. (This meant that if $CH_{\mathrm{source}}$ was Fz and $CH_{SO}$ was Cz, the difference would be 1, and if $CH_{\mathrm{source}}$ was Cz and $CH_{SO}$ was POz, the distance would be 2). *SI Appendix*, Table S1 presents the coefficients and related *P* value identified by the LME model. Also, we added SO amplitude to the model to test the effect of this predictor on the height of outflow peaks. Adding SO amplitude did not improve the model, implying that there was no relation between SO amplitude and peaks of information outflow (*P* value >0.05).

Based on LME modeling outcome, there were significant linear effects of the SO channel (*P* value <0.05), distance between the SO channel and the source ($D_{CH_{\mathrm{source}},CH_{SO}}$), and phase (pre/postpeak) on the height of outflow peaks (*P* values <0.05), with $D_{CH_{\mathrm{source}},CH_{SO}}$ having the largest linear effects (higher coefficients). The negative coefficient of $D_{CH_{\mathrm{source}},CH_{SO}}$ demonstrated that information outflow increased with proximity of the sources to the SO channel. For example, for an SO detected at the Fz channel, the lowest peak of causal information outflow was found in the POz channel, whereas moving closer to the Fz channel, the peak of the outflow increased, with highest outflow at Fz itself. The positive coefficient of the SO channel indicates that the position of the SO (anterior versus posterior) modulated the height of the outflow, where SOs in the anterior channels may produce lower outflow in all sources compared with SOs in more posterior regions. Additionally, the negative coefficient of peak phase (i.e., being a pre- or postpeak) indicates that prepeaks are expected to have greater outflow than postpeaks.

## Causal Information Flow from Sources to Sinks during SOs.

**Fig. 2.**
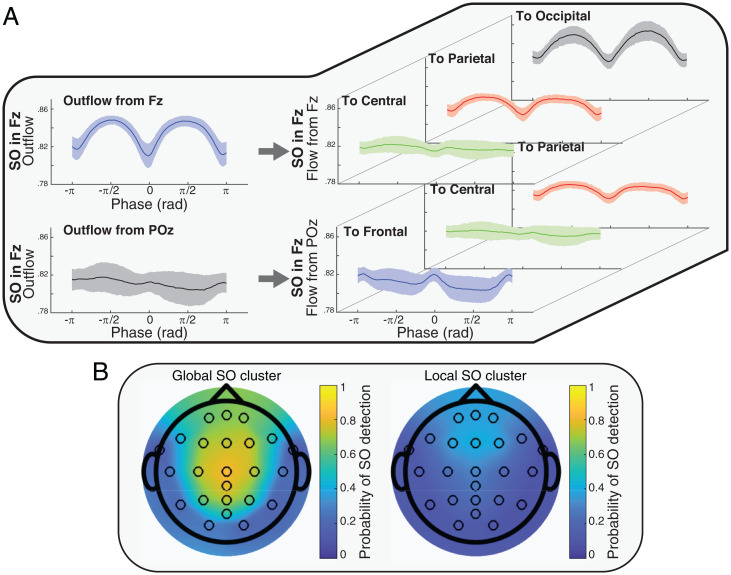
Examples of outflow and flow quantifiers and probability of the detected SOs in Global and Local clusters. (*A*) Examples of information flow from source to sink (CH→R quantifier). The *Left* plots show the total outflow from the specified source and with the presence of an SO in the specified channel (samples from Fig. 1*B*). The *Right* plots represent the portions of the flow from the source to the different sinks. For example, the *Top Left* plot shows the outflow from Fz as the source when SOs were in the Fz channel, and the three *Top Right* plots represent portions of that flow at sink. By considering the topology of the SO channels, sources, and sinks, the results suggest a relation between the distance of sink to SO channel and the peaks of information flow. (*B*) Global and Local SOs derived by the clustering method (Cluster 1: Global SOs, Cluster 2: Local SOs). The colors represent the density of the SOs in each channel.

In the previous section, we showed that the SO-related information outflow from a source was significantly affected by the distance between the SO channel and the source, where sources closest to the SO had the largest peaks. To analyze how information flow depended on sender and receiver, we next focused on source–sink pairs. Causal information flow from each source to each sink (CH→R quantifier, where Fz→P had Fz as source and parietal as sink) was computed over all SOs. For each SO channel, we averaged all CH→R phase series in each source–sink pair within each subject. Fig. 2*A* presents samples of CH→R phase series variation from two of the sources (Fz and POz) to three sinks separately (central, parietal, and occipital with Fz source and frontal, central, and parietal with POz source), all cases with SO channel in Fz (*SI Appendix*, Figs. S3–S6 provides comprehensive results).

Observing the CH→R quantifier values in Fig. 2*A* (and *SI Appendix*, Figs. S3–S6), we noticed that there was a relation between distance of the sink to the SO channel ($D_{R_{\mathrm{sink}},CH_{SO}}$) and height of the flow peaks. This suggested that the causal information flow (CH→R) could be modulated by which source–sink pair was considered, the distance of source–sink pairs to the SO channel, and the distance between the source and the sink in each pair. To investigate these potential effects, an LME model was fitted to the CH→R values in the peaks of the flow (pre- and post-SO trough), with the following linear predictors: SO channel, source–sink pairs, distance of source–sink pairs to the SO channel ($D_{CH_{\mathrm{source}},CH_{SO}}$, $D_{R_{\mathrm{sink}},CH_{SO}}$), and distance between source–sink pairs ($D_{CH_{\mathrm{source}},CH_{R_{\mathrm{sink}}}}$).

The outcome of our LME model (*SI Appendix*, Table S2) demonstrated a significant linear effect of $D_{R_{\mathrm{sink}},CH_{SO}}$ and $D_{CH_{\mathrm{source}},CH_{SO}}$ on the height of the flow peaks. Also, there was a significant effect of peak phase (pre- or posttrough), where peak height in the pretrough phase was larger than in the posttrough phase (coefficient <0). In contrast, we found no statistically significant linear effects of SO channel, source, or distance between source–sink pairs on the causal information flow peak height. The coefficient values showed different magnitude and direction of the effect in each predictor. The positive coefficient of distance between sink and SO channel and negative coefficient of distance between source and SO channel revealed that sources closer to the SO channel (less distance) and sinks farther from the SO channel (larger distance) had a higher peak of information flow in comparison to other compositions. For example, for an SO in the Fz channel, sources closest to the SO channel and sinks farthest from the SO channel (e.g., Fz→O) have the highest information flow, suggesting that brain communication during the SO is highest at locations far from the SO, and distance between SO channel to the source–sink pair impacts causal flow.

Our results support the following:

• Sources closer to the SO channel send the greatest magnitude of information ($D_{CH_{\mathrm{source}},CH_{SO}}$ coefficient for flow peaks <0).
• Sinks farther from the SO channel receive the greatest magnitude of information ($D_{R_{\mathrm{sink}},CH_{SO}}$ coefficient for flow peaks >0).
• The distance between the sink and the SO channel shows the strongest effect on information flow compared with all other predictors (absolute value of $D_{R_{\mathrm{sink}},CH_{SO}}$ coefficient for flow peaks ≫ absolute value of other predictor coefficients).

## Effect of SO Clusters on Causal Information Flow.

So far, we have shown that the impact of SOs on causal information flow depends on the distance between the source to the SO channel when considering outflow, and it depends on the distance of the sink to the SO channel when looking at specific flow between source–sink pairs. Also, channels closer to the SO channel can send more causal information flow, whereas channels farther from the SO channel can receive more causal information flow. In light of these properties, we hypothesized that the primary benefit of SO’s effect on brain information processing is to permit causal communication between remote brain regions. If this is true, it follows logically that Global SOs (SOs that propagate across a large portion of the scalp) should mediate more information flow compared to other (more localized) types of SOs. Studies investigating the spatial and temporal cooccurrences of SOs report that the majority of SOs are traveling waves, with their pattern of origin and propagation providing an outline of cortical connectivity (6). Using a cluster-based analysis of SO cooccurrences across the EEG channel manifold, prior work from our group identified three spatiotemporal categories: Frontal, Local, and Global (7), whereby Global SOs occur in most electrodes, Local SOs occur in few electrodes without location specificity, and Frontal SOs are confined to the frontal region. Along with having a greater footprint and larger amplitude, Global SOs also provided greater hierarchical nesting of thalamocortical sleep spindles, suggesting functional differences between SO categories, with Global SOs more poised to activate systems consolidation (7).

We tested the hypothesis that Global SOs should have greater causal flow than nonglobal (Local) SOs by comparing the effective connectivity in each type. We first clustered Global and nonglobal SOs using a method previously introduced by our team (7). Fig. 2*B* shows the occurrence rate of SO in each of the channels and in each of the two clusters on the scalp surface after the clustering process. Based on results from ref. 7 and our presented results in Fig. 2*B*, the two detected clusters were interpreted as Global and Local SO clusters. To test the effect of cluster on the amplitude of SOs, we first tested if there was a significant difference between amplitude of SOs in the global and local clusters. The results (*SI Appendix*, Fig. S7) showed significant differences in the clusters in each channel (*P* value <0.05). Then, we tested if there was a significant relation between amplitude of SOs and height of information outflow peaks in either cluster. Our tests showed no significant relation between amplitude of SOs and peaks of information outflow. To test if there was an effect of cluster identity (i.e., being a Global or Local SO) on the height of information flow peaks, we added cluster identity (Global as 1 and Local as 2) as a fixed effect along with other used predictors in an LME model of information flow peak height.

The results of LME models for peaks of information flow from the sources to the sinks (*SI Appendix*, Table S3) showed the same effects as the previous predictors combined with a significant linear effect of clusters on the height of the flow peaks (*P* values <0.05). Negative coefficients for the cluster effect showed that peak heights were higher in Global SOs (cluster = 1) than in Local SOs (cluster = 2). We interpret this as confirming our hypothesis that Global SOs mediate larger information flow compared to other SO types.

As a complementary analysis, we tested the effect of SO-spindle coupling on causal information flow by adding coupling as a predictor to the LME model. First, we detected all spindles that had overlap with the time window of ±1 s of SOs trough. We used the same approach as ref. 29 for spindle detection. Then, we added SO-spindle coupling as a fixed effect to the LME model by considering the value of noncoupled as 1 and coupled as 2. The result of modeling peaks of causal information flow by adding SO-spindle coupling showed a significant (*P* value <0.05) positive effect of coupling (*SI Appendix*, Table S4), with the positive coefficient for the coupling effect indicating that peak heights were higher when SOs were coupled with spindles.

## LTM Improvement and Causal Information Flow during SOs.

**Fig. 3.**
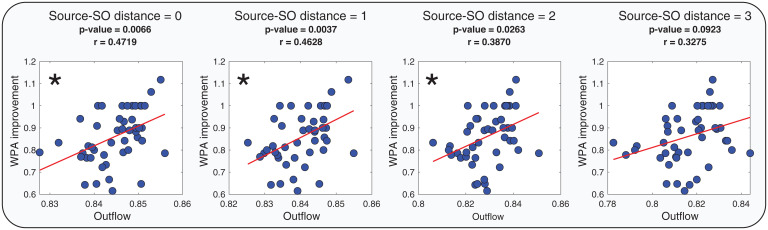
Regression tests of Outflow and WPA improvement in four different conditions of distances between the SO channel and outflow source ($D_{CH_{\mathrm{source}},CH_{SO}}=0,\;1,\;2,\;3)$. In each, we show the *P* value, the linear coefficient is reported as r, and the significant linear relationships are marked with asterisks (with *P* values adjusted to Bonferroni correction).

Next, we investigated if SO properties that we identified as crucial for information flow have functional consequences on the formation of LTMs. Based on the importance of down-to-up state transitions (30–34) matched to the postpeak in our study, we examined the relation between word-pair association (WPA) improvement and causal information flow calculated during the postpeak (peak of information flow at π/2 after the SOs trough). WPA improvement was measured as the ratio of performance after the sleep night (postsleep) to the performance before the sleep night (presleep). For each subject, we assigned the postpeak outflows to one of the four distances between the source and the SO channel ($D_{{\mathrm{CH}}_{\mathrm{source}},{\mathrm{CH}}_{\mathrm{SO}}}=0,\;1,\;2,\;3$) and then averaged within each group. We then evaluated linear relations between the outflow in each group and WPA improvement using linear regression models (Fig. 3).

Results in Fig. 3 show that memory improvement was associated with the distance between SO and sources, with a significant positive linear relation between WPA improvement and outflow when the source was close to the SO channel ($D_{CH_{\mathrm{source}},CH_{SO}}=\;0,\;1,\;2$), but not when the source was far from the SO channel ($D_{CH_{\mathrm{source}},CH_{SO}}=\;3$). $D_{CH_{\mathrm{source}},CH_{SO}}=\;0$ had the greatest r-value in the regression test, and the r-value got smaller as distance between the channels increased ($D_{CH_{\mathrm{source}},CH_{SO}}$); this can be interpreted as the outflow from sources close to SO being a better predictor of WPA improvement.

**Fig. 4.**
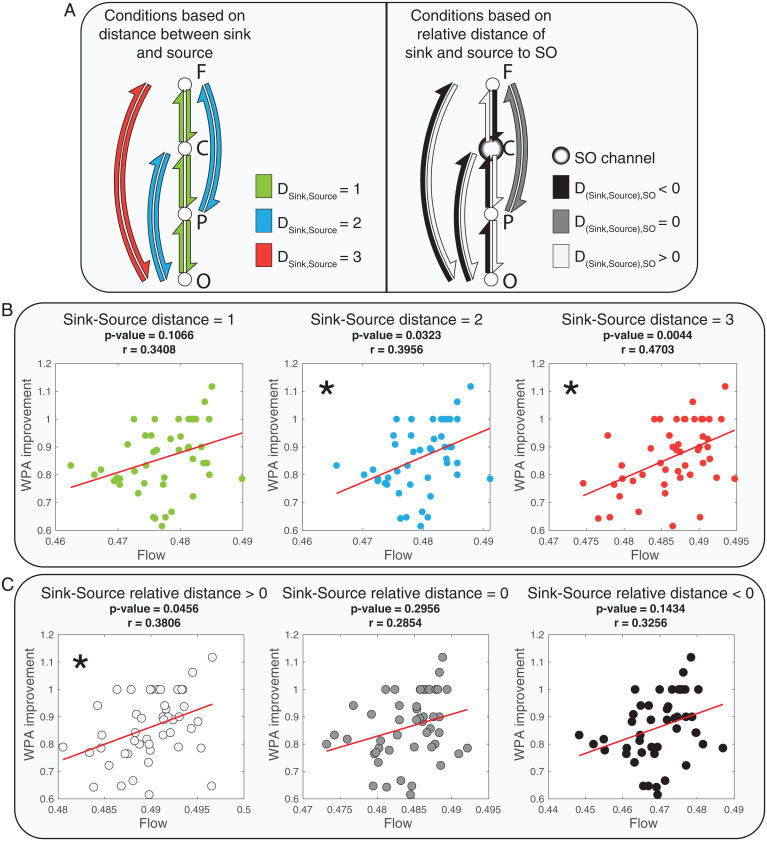
Linear relationships between flow and WPA improvement based on different distances between sinks and sources and relative distances of sinks and sources to SO channels. The asterisks mark significant linear relationships (with *P* values adjusted to Bonferroni correction). (*A*) A representation of the three conditions of distances between sinks and sources (*Left*) and an example of three possible conditions of relative distances of sources and sinks to the SO channel (*Right*, for SO channel at Cz). F, C, P and O represent frontal, central, parietal and occipital regions respectively. *Left*: Each color represents pairs of sinks and sources of information flow with equal distances. *Right*: Graph shows an example of relative distance when the SO channel is in Cz. The relative distance is greater than 0 when the source is closer to the SO channel than the sink and smaller than zero when the sink is closer to the SO channel than the source . (*B*) Correlation and regression tests of the relation between flow and WPA improvement for three conditions of distance between sources and sinks ($D_{CH_{\mathrm{source}},R_{\mathrm{sink}}}=1,\;2,\;3$). (*C*) Correlation and regression tests of the relation between flow and WPA improvement for three conditions of relative distance between sources and sinks to the SO channel.

Next, we examined how the distance between source and sink channels and the relative distance between the source and the sink to the SO channel impacted WPA improvement (Fig. 4*A*). First, we defined three possible distances between the sink and the source ($D_{CH_{\mathrm{source}},R_{\mathrm{sink}}}=\;1,\;2,\;3$) and averaged flows from sources to sinks that had the same distance. Fig. 4*B* displays the linear relation tests of WPA improvement and averaged flows in each specified distance between sink and source.

Based on the results presented in Fig. 4*B*, larger distances between sinks and sources ($D_{CH_{\mathrm{source}},R_{\mathrm{sink}}}=\;2\;\text{and}\;3$) were linked to a significant positive linear relation between information flow and WPA improvement. The r-value increased with greater distance between sink and source, suggesting a stronger linear relation between information flow and WPA improvement for long-range communications. Use of the correlation coefficient as a summary statistic—encoding the relationship between effective connectivity and memory improvement—underscores the reliability of this association as opposed to its effect size. In other words, the effect of distance between sources and sinks is on the correlation between connectivity and memory. This could reflect an increase in the effect of connectivity on memory—or a decrease in noise. This contrasts with an analysis testing for the interaction between peak connectivity and distance measures in predicting memory improvement.

Next, we examined the association of WPA improvement and flow in different relative distances from the sink and source to the SO channel. We defined the relative distances as the difference of distance between sink and SO channel and distance between source and SO channel (${\text{D}}_{{\text{R}}_{\text{sink}},{\text{CH}}_{\text{SO}}}-{\text{D}}_{{\text{CH}}_{\text{source}},{\text{CH}}_{\text{SO}}}$). Hence, relative distances greater than zero indicated that the source was closer than the sink to the SO channel. For example, if Cz was the SO channel, the relative distance of Fz source and occipital sink would be greater than zero, as ${\text{D}}_{{\text{O}}_{\text{sink}},{\text{Cz}}_{\text{SO}}}=2,\;{\text{D}}_{{\text{Fz}}_{\text{source}},{\text{Cz}}_{\text{SO}}}=1$. Fig. 4*C* shows a significant linear relation between WPA improvement and causal information flow only when the relative distance is greater than zero. Together, these results are consistent with our proposed hypothesis that long-range communication mediated by SOs plays an important role in memory consolidation. Also, to test if there is a relation between amplitude of SOs and WPA improvement, we correlated SO amplitude with and without considering the SO’s clusters. The results (*SI Appendix*, Fig. S8) showed no significant relation between amplitude of SOs and WPA improvement (*P* values <0.05).

**Fig. 5.**
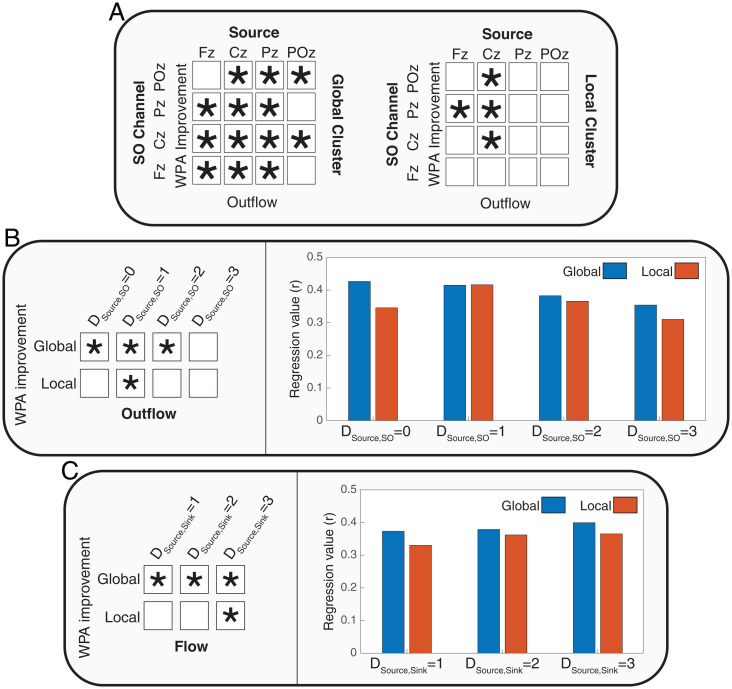
The effect of SO clusters (Local and Global) on the relation between causal information flow and WPA improvement. The asterisk shows significant linear relationships. (*A*) Summarized results of the linear relations between outflow from the source and SO channel combinations for Global and Local SO clusters (with *P* values adjusted to false discovery rate). Detailed results are presented in *SI Appendix*, Figs. S9 and S10. (*B*) Results of correlation and regression tests for four distances between source and SO channels ($D_{CH_{\mathrm{source}},CH_{SO}}=1\;to\;4$). *Left*: Presents the conditions in which there was significant linear relation between outflow and WPA improvement (with *P* values adjusted to Bonferroni correction). *Right*: Shows the r-values of regressions in different conditions of distances of sources and SO channels in the two clusters. (*C*) Correlation and regression tests for three distances between sources and sinks ($D_{CH_{\mathrm{source}},R_{\mathrm{sink}}}=1,\;2,\;3$) (with *P* values adjusted to Bonferroni correction).

A final test of this hypothesis investigated whether Global, but not Local, SOs mediated the relation between causal information flow in SOs and WPA improvement. We first measured outflow in the postpeak SO phase within Global and Local SO clusters and calculated significance levels of regressions between outflows and WPA improvement, shown in Fig. 5*A* (for the detailed results, refer to *SI Appendix*, Figs. S9 and S10). We found a greater number of channels with a significant linear relation between their outflow and WPA improvement in the Global cluster compared with the Local cluster (4 combinations of source and SO channel in the local cluster and 13 combinations of source and SO channel in the Global cluster; Fig. 5*A*).

Next, we averaged the outflows based on the distances between sources and SO channels in each cluster and examined correlations with WPA improvement. Given our prior result that short distances between source and SO channels have the highest information flow, we expected that global clusters with shorter distances between these channels would show the strongest associations between outflow and memory improvement. Indeed, our results showed significant relations between outflow in the global cluster when the distances between SO and sources were smaller than 4. In the local cluster, consistent with the reduced footprint of SOs in this cluster, we found a significant relation between outflow and WPA improvement only when the distance between SO and source was equal to 1 (Fig. 5*B*). Furthermore, r-value comparisons showed that the best condition (greatest r-value) for modeling WPA improvement was in the smallest distance between SO and source and for SOs in the global cluster.

We then conducted the same analysis between sink and source distances in each cluster. In each of the clusters, we calculated and averaged flows postpeak across distances between sink and source ($D_{CH_{\mathrm{source}},R_{\mathrm{sink}}}=\;1\;\text{to}\;3$). We found (Fig. 5*C*) a significant linear relation between flow and WPA improvement for all sink/source distances in the global cluster. Conversely, in the local cluster, there was a significant relation between flow and WPA improvement only when sink and source were at the farthest distance ($D_{CH_{\mathrm{source}},R_{\mathrm{sink}}}=3$). In sum, these results demonstrate that the best condition for modeling WPA improvement based on information flow is during global SOs across long-range neural channels.

## Discussion

In this work, we investigated temporal and topographical properties of the sleep SO by measuring effective connectivity during the SO phase, which establishes directional causality of information flow. We estimated information outflow from individual channels and flow between sources and sinks. First, consistent with prior findings, we found that information outflow at the SO trough and in non-SO windows are similarly dampened. Within this state of reduced communication, however, we identified two peaks of large-scale outflow before and after the SO trough. The direction and magnitude of the outflow peaks depended on the topographical position of the SO, dramatically decreasing with farther distances from the SO origin. Next, we considered source and sink quantifiers and determined that sources closer to the origin of the SO were the biggest senders of information, whereas sinks farthest from the SO were the highest receivers. Cluster analysis confirmed that compared to Local SOs, Global SOs mediated greater information flow. Taken together, our effective connectivity findings suggest that despite the generally disconnected state of NREM communication, SOs facilitate two bursts of long-range causal communications across brain areas. As the goal of the study was to determine how the brain organizes network activity to support memory, we tested the temporal and topographic conditions of NREM sleep that predict overnight memory improvement. Our results show that memory improvement is predicted by conditions that maximize the distance between sink and source channels relative to the SO origin, specifically during global SOs. Our findings are consistent with the notion that SOs provide the temporal framework for systems-level communication, which is a necessary condition for hippocampal, episodic memory consolidation.

### GPDC as a Granger Causality Measure.

Based on its mathematical definition, Granger causality can estimate the causal information flow between different time series, which is then interpreted as effective connectivity. The main aspect that differentiates effective connectivity from functional connectivity is its ability to quantify relative influences that are asynchronous. Therefore, Granger causality can find “predictive causality” (35) and identify the cause-effect relations with constant conjunctions (36). Within this concept, GPDC was used in this study to model the causal information flow between different regions that could be found in sleep EEG signals. GPDC as a generalized form of PDC can distinguish indirect and cascade connections from direct connections, based on partial coherence notation (28). PDC (and GPDC) will show propagation of information flow only when there is phase difference between sink and source, thus producing null information flow as a result of factors that have no phase difference, such as volume conduction. In practice, volume conduction can have some minor influence on GPDC calculations, such as increasing the noise level. These influences are not critical (37), especially for our study, in which we averaged the GPDC values over many samples (SOs).

With respect to the potential confound introduced by SOs being large amplitude objects, it is important to note that GPDC leverages weighted averaging to bypass amplitude difference between signals. Although greater amplitude in EEG signal can potentially represent enhanced synchrony in the neuronal activity giving rise to the signal, and hence can potentially be a source of information, the very definition of Granger causality between a sender and a receiver of an information pair involves a balance of the amplitude between signals, thus accounting for amplitude and rendering GPDC not sensitive to high amplitude effects.

### SOs Shape the Causal Communication Network in NREM Sleep.

Oscillatory activity has been linked to neural firing rate and spiking activity via multi- and single-unit recording and intracranial EEG (38). Although higher firing rate and spiking activity has been interpreted to signify greater potential outflow of information (39), the precise nature of the SO-triggered information flow has yet to be identified. Nir et al. (2011) (38) reported that the mean firing rate of units within brain regions (e.g., orbitofrontal cortex, anterior cingulate, supplementary motor, parietal cortex, parahippocampal gyrus, and hippocampus) increased up to 200% 500 ms before and after the SO trough and decreased by 40% within the SO trough in comparison with the mean firing rate in NREM (N2 and SWS). In the current work, we also found two peaks of information outflow (pre- and post-SO trough); however, the timing and phase of the outflow peaks in our study (±π/2 and about ±250 ms from the SO trough) did not match the peak firing rate reported by Nir et al. (2011) (38) (±π and about ±500 ms from the SO trough), suggesting that there are other components and conditions that affect causal information flow in addition to spiking activity. While spiking activity can represent the abundance of information in a source, availability of the sink to receive such information is a necessary condition to active communication, like causal information flow. Thus, despite a peak in neuronal firing rate 500 ms before and after the SO trough, our data suggest that the best condition for information flow between sources and sinks is found closer to the SO trough, at about ±250-ms delay.

Prior studies have demonstrated that brain communication is significantly modulated by the transition from wake to sleep. Using transcranial magnetic stimulation to probe propagation patterns across wake and sleep stages, Massimini et al. (18) and Pigorini et al. (40) showed the lowest levels of brain connectivity during NREM sleep. Furthermore, effective connectivity analysis using functional MRI and simultaneous EEG recordings has described N2 as an unstable network and SWS as a stable network (41). Together with our current findings of equivalently low levels of information outflow during SO troughs and non-SO windows during NREM, one emergent hypothesis is that causal communication across brain regions during NREM sleep is generally poor, but punctuated by bursts of selective high-communication events mediated by SOs.

### Causal Information Outflow Depends on Distance from SO Channel.

Our results demonstrate several key properties of the SOs that contribute to their communication profile. Along with time and phase of the SO gating causal information outflow, our results demonstrate that SOs enable information flow with topographic specificity as well. In particular, the distance between source and SO channels had the strongest effect on peak height (i.e., amount of information flow). Thus, the closer the source or information sender is to the SO channel, the higher the information outflow. These peaks were not identical before and after the SO trough, suggesting that brain communication differs during the down-to-up transition compared with the up-to-down transition of the SO. One potential explanation for these differences may be that we combined SOs of different types, including single and sequential SOs (42), wherein sequential SOs have pre- and post-SO peaks that are temporally linked with each other. In these cases, the prepeaks may be influenced by the preceding SO and the current SO, whereas posttrough peaks would be affected only by the current SO. In agreement with our findings, studies have shown functional differences between pre- and post-SO activity, including greater synchronization between SOs and spindles (43, 44) and more efficient cueing during targeted memory reactivation (TMR) during the post-SO trough peak (34). Thus, our data support the notion that there are functional and effective connectivity differences between SO peaks but that further research is needed to tease apart properties of single or sequential SOs. Lastly, by modeling the outflow peaks, we showed a significant effect of the SO channel, with the positive coefficient demonstrating an increase in the height of outflow from anterior to posterior regions. These results are likely due to the presence of significantly fewer SOs in posterior regions, suggesting the intriguing possibility that total information flow is not higher in the posterior channel in general, but rather that during the few posterior SOs that do occur, there is a higher amount of information outflow than in other areas. Future research may determine why outflow increases as the number of detected SOs diminishes.

### SOs Facilitate Causal Communication between More Distant Regions.

The SO has also been described as a global traveling wave that recruits the entire cortical network (45). We therefore probed information flow by considering source and sink channels independently and together as quantifiers and discovered that regions farther from the SO channel are more engaged in receiving information. This suggests that global brain communication during the SO may be organized with local areas near the SO sending more information and local areas farther from the SO receiving more information. As such, brain areas engaged in high causal information outflow (i.e., sending information) have a low chance of simultaneously receiving information. Given these results, we hypothesized that the effect of distance between sinks and sources and greater causal communication between farther regions should be specifically accentuated in the case of global SOs, which involve a greater number of scalp locations (7). We tested this hypothesis by using Global or Local SOs as predictors in our model and demonstrated a significant effect of SO cluster on the height of the flow peaks, with Global SOs having higher peak heights than Local SOs.

### SOs as a Facilitator for LTM.

One leading hypothesis of memory consolidation suggests that encoding networks get reactivated during sleep (46), specifically during NREM sleep (47). Given the near-infinite diversity of the encoding networks, memory reactivation requires both short- and long-range information transfer across a wide range of brain areas. It is well established that SOs provide temporal coordination for memory-related brain activity during systems consolidation (47), and a greater effect of TMR has been shown when cues are coupled with the down-to-up phase of the SO (31, 34), similar to the postpeak in the current results. However, NREM is a sleep period generally characterized as showing a breakdown in connectivity with increasing depth (18), which would not be conducive to reactivation.

Our results pose a potential solution to this seeming paradox, with Global SOs providing the temporal-topographical event framework whereby long-range information flow increases dramatically compared to the relatively local activity patterns of the rest of the sleep period. Specifically, as memories get reactivated in cortical-hippocampal networks, traveling Global SOs mediate systems-level communication across all source–sink pairs, even for sources far from the SO origin and close sink and source pairs. In agreement with this finding, for Local SOs the relation between causal flow and episodic improvement was limited to sources closest to the SO channel and long-distance pairs of sinks and sources.

By describing the space-time dynamics of sleep oscillations, we can noninvasively acquire a window on the coordination and brain processing patterns of basic psychological function as well as on cognitive disorders or conditions that currently lack a mechanistic connection between their understood origins and their cognitive manifestations. Intriguingly, we have shown that SOs coupled with sleep spindles increase causal information flow. Future investigations should test how nested oscillations (e.g., SO–spindle complexes) promote greater information flow and their functional relevance for memory consolidation.

### Limitations and Suggestions.

Limitations of this study include combining all SOs in the calculation of causal information flow, ignoring distinctions between single or sequential SOs, which may have affected the results in pre- versus postpeaks. Our Granger causality analysis was applied to sensor-level data (EEG signals), which is clearly not optimal when talking about directed connectivity among neuronal sources. In principle, one could use a source reconstruction technique to create a set of virtual electrodes and then repeat the Granger causality analysis in source space. This would lend greater validity to our conclusions in relation to the underlying cortical connectivity. Also, we have referred to GPDC (a Granger causality analysis) as effective connectivity to emphasize its causal nature. However, strictly speaking, Granger causality is a directed form of functional connectivity because it rests upon statistical associations. A more complete analysis would be dynamic causal modeling of complex cross spectra, effectively parameterizing a neural network in source space and then finding the effective connectivity that best explains the Granger causality. The advantage of this kind of modeling is that it can resolve recurrent effective connectivity. In addition, we focused on frontal to occipital brain regions, ignoring temporal regions, which may affect interpretation of results. For each region, we considered three EEG channels that contributed to a region’s estimate. It is possible that increasing the number of channels included in each region (i.e., increasing scalp resolution) may produce more robust results. Also, considering a larger number of regions (e.g., fronto-central, centro-parietal, and parieto-occipital) may improve the efficiency of modeling the relation between areas. In this study, the simplest approach for attributing values to the distance of channels and regions was adopted (discrete linear values). The effect of these distances in our estimates could alternatively be modeled with real-world values of the distances across the brain cortex. GPDC as a Granger causality estimator—consistent with other methods in this family—represents effective connectivity in the frequency domain. In an effort to reduce complexity, we chose to average the effective connectivity values, supported by the lack of mechanistic reasons, to preferentially concentrate on a specific a priori frequency range for directional connectivity estimates. Future studies should address potential differences in effective connectivity profiles when GPDC is evaluated only in specific frequency bands, possibly concentrating on ranges that are known for their physiological relevance and interaction with SOs, such as sigma.

## Materials and Methods

### Data.

Overnight sleep was measured in 59 healthy adults (20.5 ± 2.57 y, 26 females) with no history of psychological and neurological problems. All participants signed informed consent. This study was approved by the University of California, Riverside Human Research Review Board. EEG signals were acquired using a 32-channel cap (EASEYCAP GmbH) with Ag/AgCI electrodes placed according to the international 10–20 System at a sampling rate of 1,000 Hz. Twenty-two EEG channels were used for EEG signal recording, and the others were used for reference, ground, and other biosignals, including electromyography, electrooculography, and electrocardiography. EEG signals were rereferenced to the contralateral mastoid (A1 and A2) and down-sampled to 256 Hz after the recording. Raw data were visually scored in 30-s epochs into Wake, Stage 1, Stage 2, SWS, and REM sleep according to the Rechtschaffen & Kales’ manual (48) using HUME (49), a custom MATLAB toolbox. All 22 EEG channels were used for SO detection and the clustering process. Effective connectivity was computed considering 12 EEG channels (F3, Fz, F4, C3, Cz, C4, P3, Pz, P4, O1, POz, and O2), which were filtered with a Butterworth high pass filter with stop band of 0.5 Hz and order of 46.

**Fig. 6.**
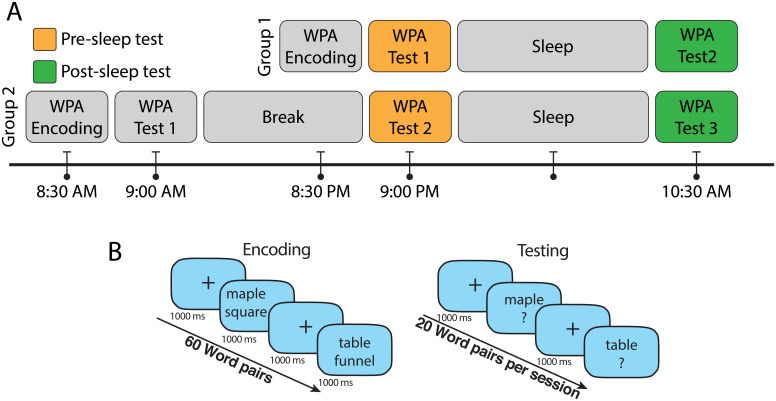
Timeline of the encoding and test sessions of WPA task and details of the encoding and test sessions. (*A*) Participants in group 1 (*n* = 26 after excluding participants who did not complete all the task sessions) reported to the laboratory in the evening and completed the encoding session of WPA and immediate testing (Test 1, presleep) at around 9 PM and prepared for sleep. They were awakened in the morning and after breakfast completed the next testing session (Test 2, postsleep) at 10:30 AM. In group 2, participants (*n* = 26 after excluding participants who did not complete all the task sessions) completed the WPA encoding and immediate testing session (Test 1) at around 8:30 AM. Then, participants left the laboratory to continue their day and came back to the laboratory to complete the second testing session (Test 2, presleep) at around 9 PM. The next morning, participants were awakened at 9 AM and after breakfast completed the last test (Test 3, postsleep) at 10:30 AM. (*B*) In the encoding session of the WPA task, 60 pairs of words with length of three to nine letters were presented vertically stacked and shown twice in random order. Each word pair was presented for 1,000 ms and followed by a fixation cross for 1,000 ms. Immediately after encoding, subjects were trained to criterion using a test in which participants were shown one word of the pair and were required to type in the associated word. Incorrect trials were repeated, and feedback was provided. The participants had to achieve 70% accuracy to finish the encoding session. The used word pairs in the encoding session were divided into three groups of 20 word pairs for the testing sessions. In each testing session, participants were shown one word of the pair and were required to type the associated word.

To investigate the relation between causal information flow and improvement in LTM, the WPA test was deployed. The subjects were randomly divided into two groups. The first group completed the WPA encoding session before a night of sleep, and the second group completed the WPA encoding session in the morning followed by a night of sleep. As we were interested in causal information flow in SOs and its effect on improvement of LTM, performance in the presleep (test 1 in the first group and test 2 in the second group) and postsleep (test 2 in the first group and test 3 in the second group) test sessions of WPA task was used, and the ratio of postsleep performance to presleep performance was considered as sleep-related WPA improvement ratio (Fig. 6).

### SO Detection.

We used an SO detection algorithm introduced by Dang-Vu et al. (50) to find SOs in Stage 2 and SWS, measuring zero-crossing points of filtered EEG signal in the 0.1- to 4-Hz range. Four criteria were used to identify an SO: 1) the wave minimum was below or equal to 80 uV; 2) the range of values between maximum and minimum voltage was at least 80 uV; 3) the time between the first and second zero crossing in the data had to be between 300 ms and 1 s; and 4) the total duration of the candidate event was at most 10 s. The time margin of detected SOs from the Fz, Cz, Pz, and POz channels was used for effective connectivity computation, and all detected SOs in all 22 EEG channels were used for the clustering process.

### Effective Connectivity Estimation.

Granger causality is an analytical definition of causality between two time series. Intuitively, according to this definition, time series $x_{1}$ Granger causes $x_{2}$ if using past values of $x_{1}$ ($t<t_{0}$) improves the prediction of the current value of $x_{2}$ ($t=t_{0}$) (26). This principle can be modeled via a multivariate autoregressive (MVAR) model. MVAR models the interactions between past values of N signals (X) in relation to the present time values ($X\left(n\right)=\left\{x_{1}\left(n\right),\;x_{2}\left(n\right),\;\ldots ,\;x_{N}\left(n\right)\right\}$) using a linear regressive model (Eq. **1**):

[1] $$X\left(n\right)=\overset{p}{\underset{k=1}{\sum }}A_{k}X\left(n-k\right)+w\left(n\right),$$

where X(n) is a vector containing amplitudes of all signals in time, n marks the time stamp at which X is being computed, and p is the model order (i.e., how many steps back in time are used to model the current values). $A_{k}$ is the matrix of MVAR coefficients, and each element $a_{ij}\left(k\right)$ represents how much the *j-*th signal at time n−k affects the i-th signal at time n. w(n) is the model’s additive Gaussian noise vector, with zero mean and covariance matrix Σ. Several measures use MVAR to estimate Granger causality–based effective connectivity, such as the Granger causality index, directed transfer function, PDC, and numerous variations of these measures (51). PDC measures the directed linear relation between two time series using MVAR coefficients in the frequency domain (Eq. **2**):

[2] $$PDC_{ij}\left(f\right)=\frac{A_{ij}\left(f\right)}{\sqrt{\sum_{k=1}^{N}{|A_{kj}\left(f\right)|}^{2}}},$$

where f is normalized frequency [0 to 0.5] Hz (here based on the sampling rate of 256 relative to actual frequency range of 0 to 128 Hz), where $A_{ij}\left(f\right)$ is the MVAR coefficients in frequency domain (Eq. **3**). In this study, the frequency is sampled at 1/128 Hz relevant to 1 Hz in actual frequency:

[3] $$A_{ij}\left(f\right)=\delta_{ij}-\overset{p}{\underset{k=1}{\sum }}a_{ij}\left(k\right)e^{-2j^{*}\pi fk},$$

for $\delta_{ij}=1$ whenever i=j and $\delta_{ij}=0$ otherwise and $j^{*}=\sqrt{-1}$.

PDC can be affected by unbalanced signals amplitude. Therefore, its generalized version (GPDC) introduces a redefinition that improves PDC’s estimations in scenarios with severely unbalanced errors caused by unbalanced amplitude scales (27). Specifically, GPDC uses elements of noise covariance matrix (Σ) to overcome this potential bias (Eq. **4**):

[4] $${\bar{\pi }}_{ij}\left(f\right)=\frac{\frac{1}{{\text{Σ}}_{ii}}A_{ij}\left(f\right)}{\sqrt{\sum_{k=1}^{N}\frac{1}{{\text{Σ}}_{kk}{}^{2}}{|A_{kj}\left(f\right)|}^{2}}},$$

where ${\bar{\pi }}_{ij}\left(f\right)$ is the matrix of causal information flow (GPDC), in which the *j*-th column estimates causal information outflow from the *j*-th signal to all other signals, while N counts the total number of signals considered. The values are normalized over each column separately; therefore, outflows in each column are comparable only within the specific column.

For each considered SO event in each of Fz, Cz, Pz, and POz channels, the time interval of 1 s before to 1 s after the negative trough of the SO was considered for the analysis. To compute causal information flow during SOs, GPDC was computed in sliding windows for each SO separately, which allowed us to follow causal information flow changes over time. The window length (w) was 0.5 s to maintain a relative stationarity of EEG signals, and stride was 7.8 ms. Therefore, we estimated 193 GPDC three-dimensional tensors over the time course of each SO. Akaike information (52) was used to choose the appropriate model order p, resulting in p=13 selected for all the windows. To reduce the complexity of computations and have more robust results, the GPDC tensors sized $N_{x}N_{x}N_{\mathrm{Frequency}}$ were averaged over the frequencies to construct the GPDC matrix π(i,j). Also, to reduce variance, causal information outflows from a given channel (Fz, Cz, Pz, and POz) to all channels in other areas, including frontal, central, and parietal, were averaged. Fig. 1*A* represents the process of estimating the outflow to each area. Twelve channels of EEG signals (N = 12) were considered for computing GPDC matrices (F3, Fz, F4, C3, Cz, C4, P3, Pz, P4, O1, POz, and O2). Fz, Cz, Pz, and POz were considered sources of causal information in estimating effective connectivity, and outflow from these selected central channels to all 12 channels was quantified for causal information flow. We defined two types of outflow quantifiers, one that combined the outflow to all channels in regions different from the one that included the source and one that concentrated on a given sink region. Their respective equations are

[5] $$\begin{array}{ll} CH_{\mathrm{Outflow}}^{}=\underset{i\in D}{\sum }{\left(\pi \left(i,CH\right)\right)}^{2}, & D=\begin{array}{l} \{\mathrm{all channels excluding channels in the}\\ \mathrm{same region as}\;CH\} \end{array}\\ CH\to R=\underset{i\in R}{\sum }{\left(\pi \left(i,CH\right)\right)}^{2}, & R=\left\{\mathrm{all channels in the}\;R\;\mathrm{region}\right\}. \end{array}$$

Here, CH is one of the four source channels, and R is one of the sink regions (frontal [F] including [Fz, F3, and F4] channels, central [C] including [Cz, C3, and C4] channels, parietal [P] including [Pz, p3, and P4] channels, and occipital [O] including [POz, O1, and O2] channels). These quantifiers were computed for each window of SOs and averaged in different conditions, including SOs in each source channel separately and different pairs of source channel and sink region.

π(i,j) matrices were calculated for each SO in Fz, Cz, Pz, and POz channels as the source of causal information flow channels. By using the sliding window technique, for each 500-ms window, the quantifiers ($CH_{\mathrm{Outflow}}^{}$ and CH→R) for all four source channels and sink regions were calculated (Fig. 1*A*). The resulting values were assigned to the central time of their respective window. Therefore, for each SO in a source channel and each quantifier, there were 193 values between 750 ms before to 750 ms after the SO trough. For each participant, each quantifier was averaged over all SOs in each of the Fz, Cz, Pz, and POz channels separately, producing a time series per each quantifier per channel.

To access the quantifiers’ variation over the SO’s phase, we used nonlinear resampling in three steps. In the first step, the phase of each SO was estimated using the Hilbert transform between 1 s before and 1 s after the SO trough (whose phase was assigned as zero as it is represented in Fig. 1*B*). In the next step, we selected a valid time range by including only the times in which the phase increased monotonically between −π and π. In the last step, we generated a phase series in the valid time range increasing from −π to π with a fixed step at π/64 by resampling the quantifier within the valid times. Although the proposed method starts with the SO trough as the timing reference, this referencing is immediately lost after resampling from the time to the phase domain. Thus, any exact timing reference is removed in favor of matching all pre- and posttrough peaks to their phases. For example, the computed quantifiers of the windows centered at positive peak of EEG before the SO troughs in Fz channel have been averaged such that their phases are the same (−π).

### Clustering.

The same approach as in ref. 7 was used to cluster SOs into global and nonglobal clusters. Briefly, for each detected SO in each of the 22 channels, a binary vector V with the length of the number of all channels was constructed (Eq. **6**):

[6] $$V=\left[c_{1},\;c_{1},\ldots ,\;c_{22}\right],$$

where $c_{j}$ was assigned 1 if there was an SO in the *j*-th channel in the time range of ±400 ms of the SO’s trough time in the *i*-th channel and 0 if there was not an SO in that time margin in the *j*-th channel. Next, a K-means algorithm with Hamming distance was run to categorize all vectors into two clusters (K = 2). The K-means algorithm was run with 200 repetitions, minimizing within-cluster distance, in both stage Stage 2 and SWS.

### Statistical Analysis.

An LME model was used to model the GPDC causal information flow peaks and investigate the effect of different variables, including source channel, sink region, etc. The LME is an extension of linear regression models for data that are collected and summarized in groups (53). LME models describe the relation between a response variable and the independent variables, with coefficients that can vary with respect to one or more grouping variables. Each participant was considered as a random effect, and each variable was considered as an independent fixed effect. After an LME model was fit to the data, multiple values were considered, including the coefficients of the model and *P* value related to each parameter. The *P* value of each fixed effect was interpreted as a significant linear effect of the variable on the model. To investigate the predictors as independent fixed effects, we assigned numbers to predictor values using a linear approach. Numbers 1 to 4 were used for the anterior channel or region (Fz or frontal region) to the posterior channel or region (POz or occipital region). The same numbers were used to represent the distance between channels and regions. Also, to model the effect of the phase on the amplitude of the peaks, the prepeak of the flow was identified with the value 1, and the postpeak of the flow was marked with 2.

ANOVA was used to compare quantifier values in each peak and the non-SO windows. Also, one-way ANOVA was performed to test significance between different time windows during SOs and non-SO, and post hoc analysis was applied to identify windows that were significantly different from the non-SO windows.

A linear model was used for a correlation and regression test to investigate the linear relation between WPA improvement ratio and causal information flow in different types of conditions. Three types of conditions were considered to group and average the causal information flow in each condition and fit the linear model using the averaged flow as the predictor and WPA improvement ratio as the dependent variable. In the first type of condition, the distance between the SO and the source channel (${\text{D}}_{{\text{CH}}_{\text{source}},{\text{CH}}_{\text{SO}}}$) was considered as the condition in which the distance could be 0 to 3 (total 4 condition groups). The distance between the source channel and the sink region ($D_{CH_{\mathrm{source}},R_{\mathrm{sink}}}$) was considered as the second condition type with a value of 1 to 3 (total 3 condition groups). The third condition type was considered as the relative distance between the sink and the source with respect to the SO channel, which was defined as ${\text{D}}_{{\text{R}}_{\text{sink}},{\text{CH}}_{\text{SO}}}-{\text{D}}_{{\text{CH}}_{\text{source}},{\text{CH}}_{\text{SO}}}$, and condition groups were defined as positive, negative, and zero values (total 3 condition groups). The positive values represent the condition where the source is closer to the SO channel than the sink to the SO channel, and the negative value shows the opposite condition. In all the statistical tests, mean ±2 SD was considered as the outliner boundary, and data outside of this margin were excluded.

## Supplementary Material

Supplementary File

## Data Availability

Data have been deposited at GitHub (54).
