# Supplementary material for: Slow oscillations promote long-range effective communication: The key for memory consolidation in a broken-down network
Source: Proc Natl Acad Sci U S A. 2022 Jun 22;119(26):e2122515119. doi: 10.1073/pnas.2122515119 (PMC9245646; doi:10.1073/pnas.2122515119)
Supplement: Supplementary File [file pnas.2122515119.sapp.pdf]

Supplementary figures and tables

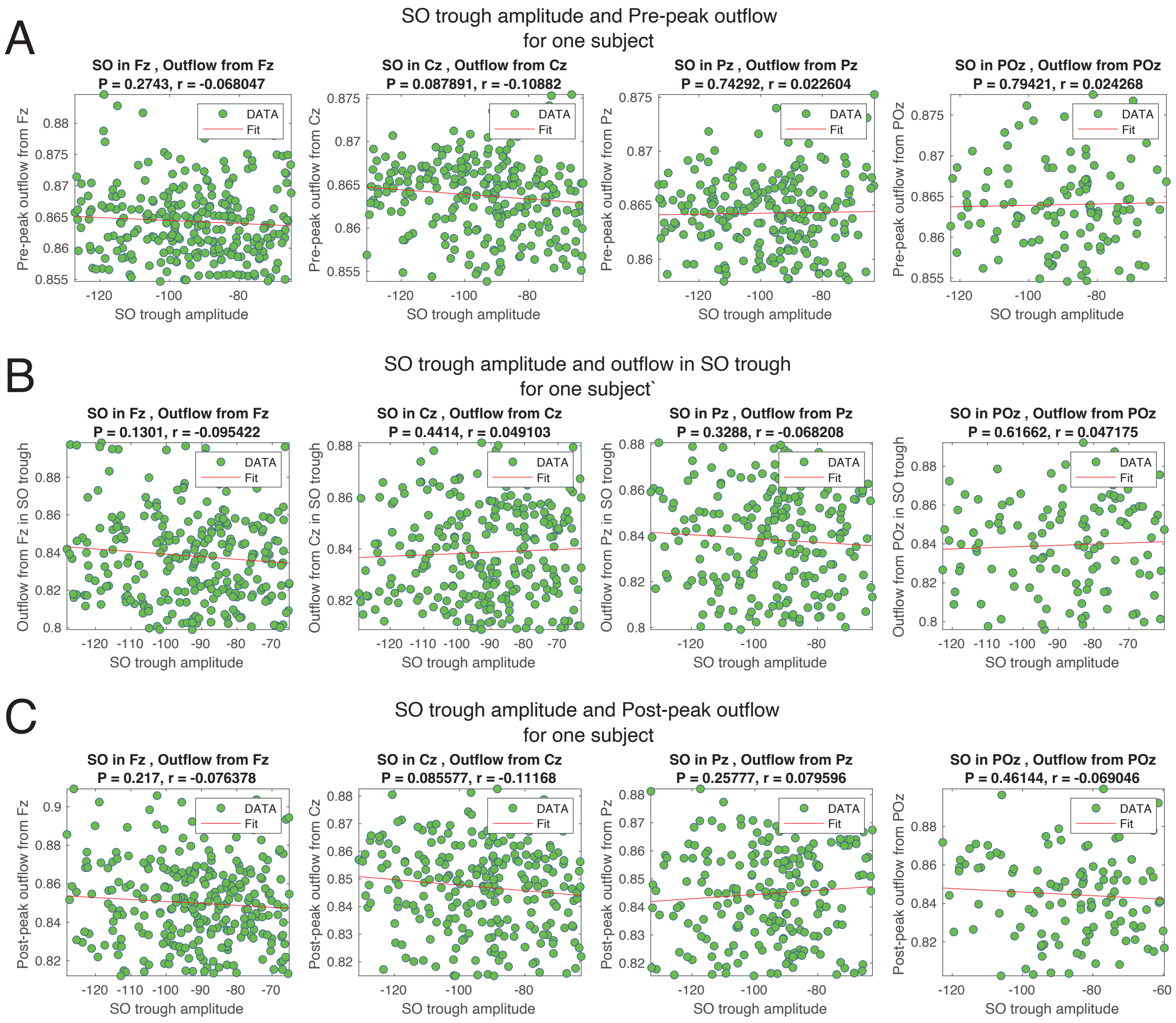

Figure S. 1 Correlations between SO amplitudes and information outflow using all SOs of an example subject in each of the channels. A) Correlations between SO trough amplitudes and pre-peak (phase = -π/2) outflow. B) Correlations between SO trough amplitudes and outflow in SO troughs (phase = 0). C) Correlations between SO trough amplitudes and post-peak (phase = π/2) outflows. No significant relations between SO amplitudes and outflows in different phases were found.

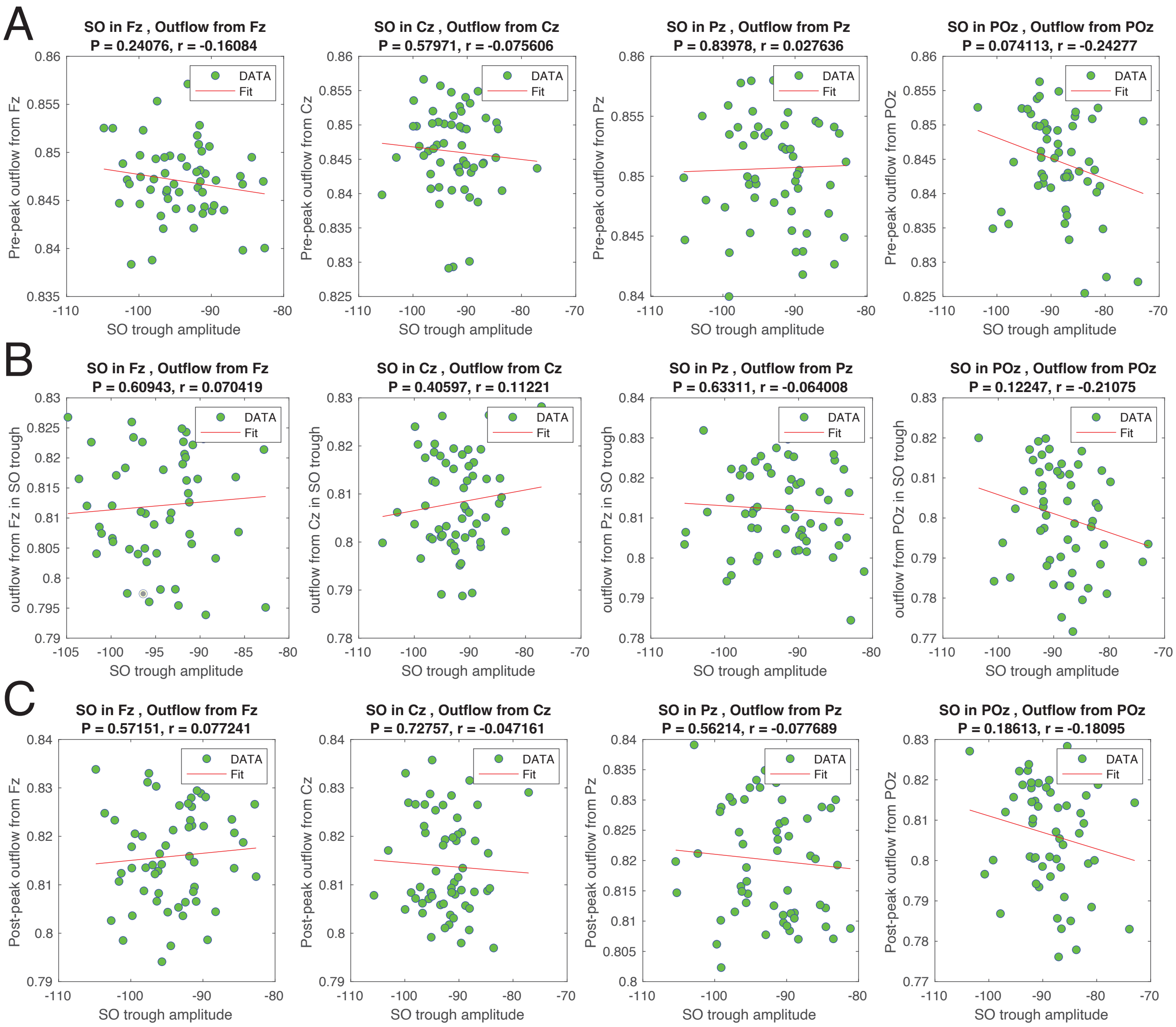

Figure S. 2 Correlations between SO amplitude averages for each subject and information outflows in each of the source/SO channels separately. A) Correlations between SO amplitudes and pre-peak (phase = -π/2) outflows. B) Correlations between SO amplitudes and outflows in SO trough (phase = 0). C) Correlations between SO amplitudes and post-peak (phase = π/2) outflows. No significant relations were found between SO amplitudes and outflows in different phases considering all the subjects.

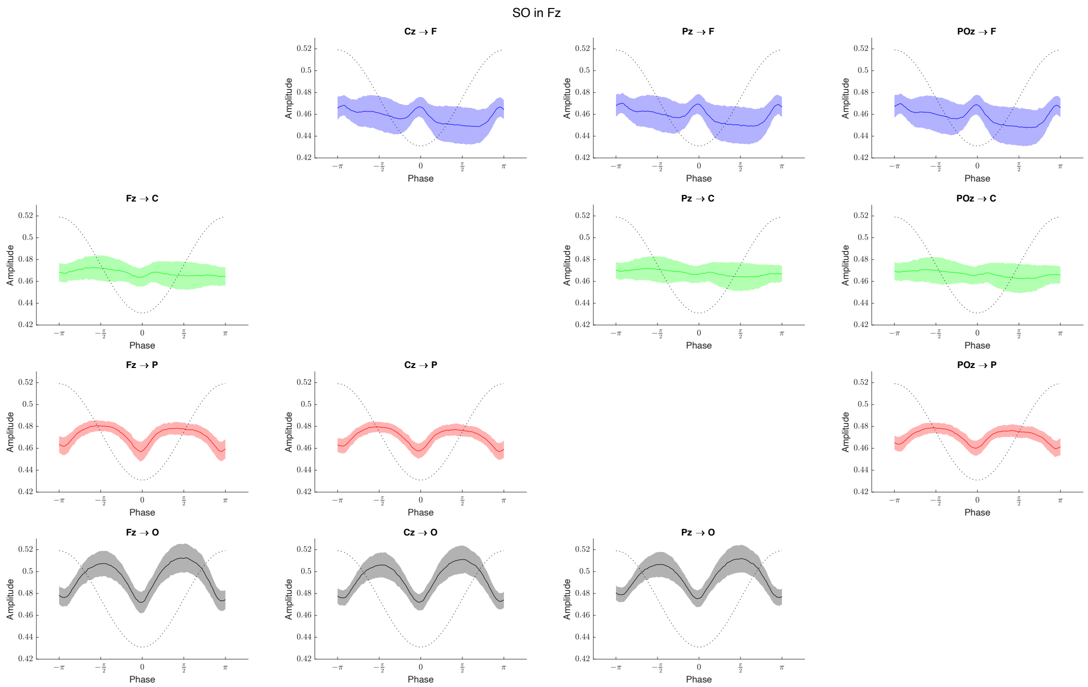

Figure S. 3 $CH \rightarrow R$ quantifiers for each of the source channel–sink region when SO channel is Fz. Each row represents the flow phase series to specific sink region. Each column represents flow phase series from each of the source channels.

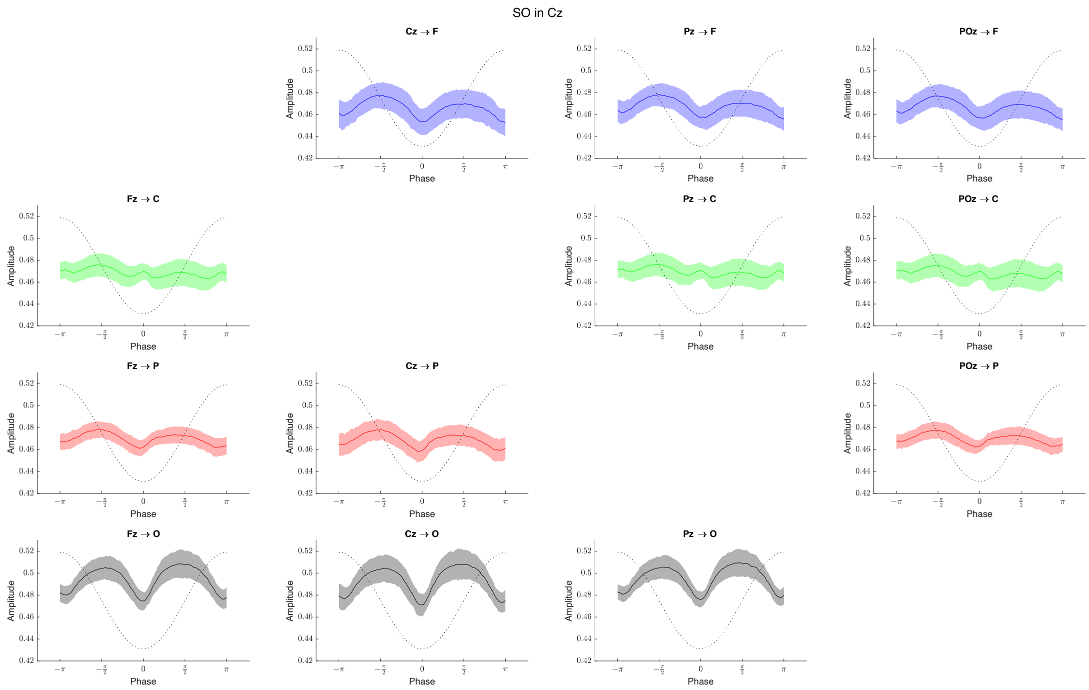

Figure S. 4 $CH \rightarrow R$ quantifiers for each of the source channel–sink region when SO channel is Cz. Each row represents the flow phase series to specific sink region. Each column represents flow phase series from each of the source channels.

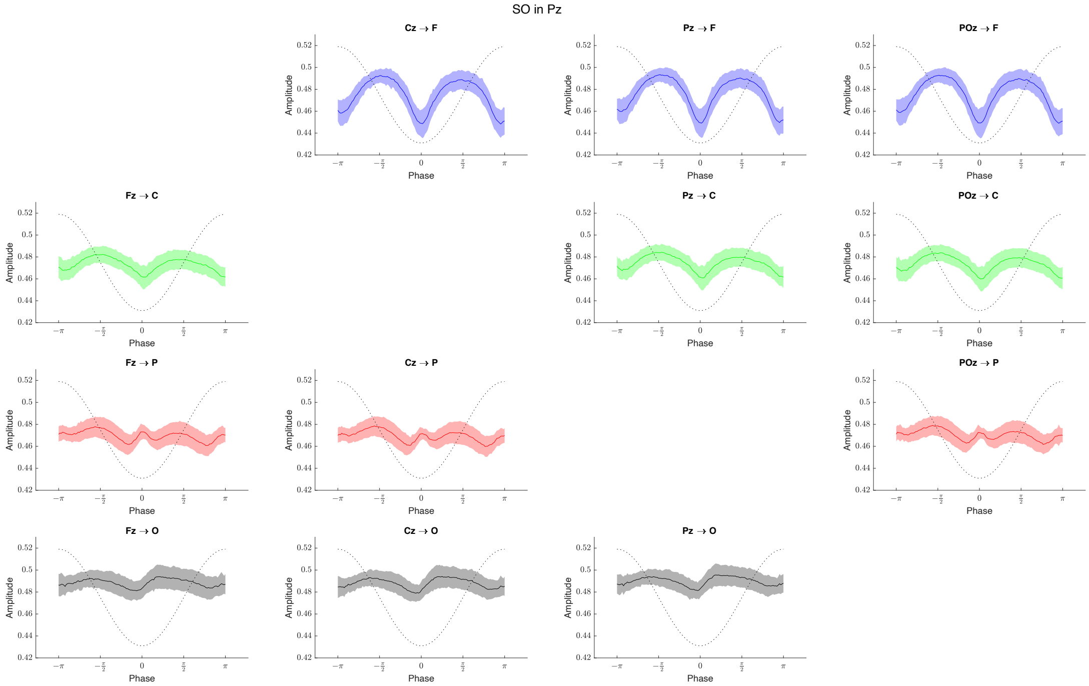

Figure S. 5 $CH \rightarrow R$ quantifiers for each of the source channel–sink region when SO channel is Pz. Each row represents the flow phase series to specific sink region. Each column represents flow phase series from each of the source channels.

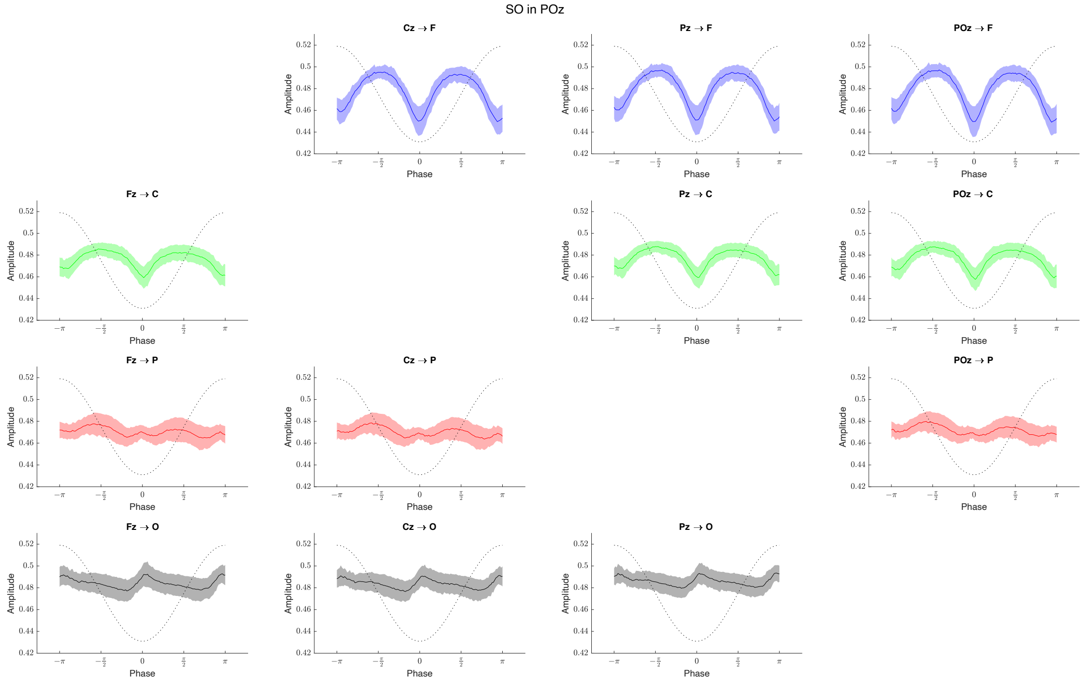

Figure S. 6 $CH \rightarrow R$ quantifiers for each of the source channel–sink region when SO channel is POz. Each row represents the flow phase series to specific sink region. Each column represents flow phase series from each of the source channels.

A

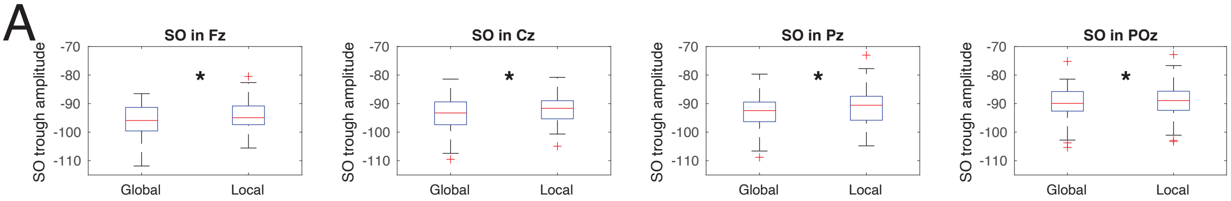

B

	Global SO: SO in Fz $Fz_{Outflow}$	Global SO: SO in Cz $Cz_{Outflow}$	Global SO: SO in Pz $Pz_{Outflow}$	Global SO: SO in POz $POz_{Outflow}$	Local SO: SO in Fz $Fz_{Outflow}$	Local SO: SO in Cz $Cz_{Outflow}$	Local SO: SO in Pz $Pz_{Outflow}$	Local SO: SO in POz $POz_{Outflow}$
Pre-peak outflow	p = 0.057069<br>r = -0.26302	p = 0.2036<br>r = -0.17412	p = 0.24215<br>r = -0.15889	p = 0.73599<br>r = -0.047418	p = 0.41727<br>r = -0.11056	p = 0.61868<br>r = -0.069271	p = 0.81823<br>r = -0.031412	p = 0.35217<br>r = -0.12787
Outflow in SO trough	p = 0.39881<br>r = -0.11716	p = 0.91324<br>r = 0.014757	p = 0.45019<br>r = -0.10201	p = 0.51648<br>r = -0.088523	p = 0.27945<br>r = 0.14706	p = 0.57818<br>r = 0.075211	p = 0.55427<br>r = -0.079255	p = 0.26871<br>r = -0.15321
Post-peak outflow	p = 0.85778<br>r = -0.024967	p = 0.061878<br>r = -0.24892	p = 0.13047<br>r = -0.20271	p = 0.22674<br>r = -0.16414	p = 0.44433<br>r = 0.10333	p = 0.87251<br>r = -0.021733	p = 0.73311<br>r = 0.045747	p = 0.1349<br>r = -0.20415

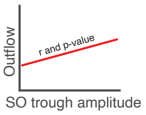

Figure S. 7 A) Distribution of SO amplitudes in each of the channels and in each of the two clusters (Local and Global). SOs in the Global clusters had greater amplitude (absolute value) than the Local cluster. B) The same analyses as Figure S.2 for SOs in each of the clusters separately. The results showed no significant relation between SO amplitudes and outflows in either cluster.

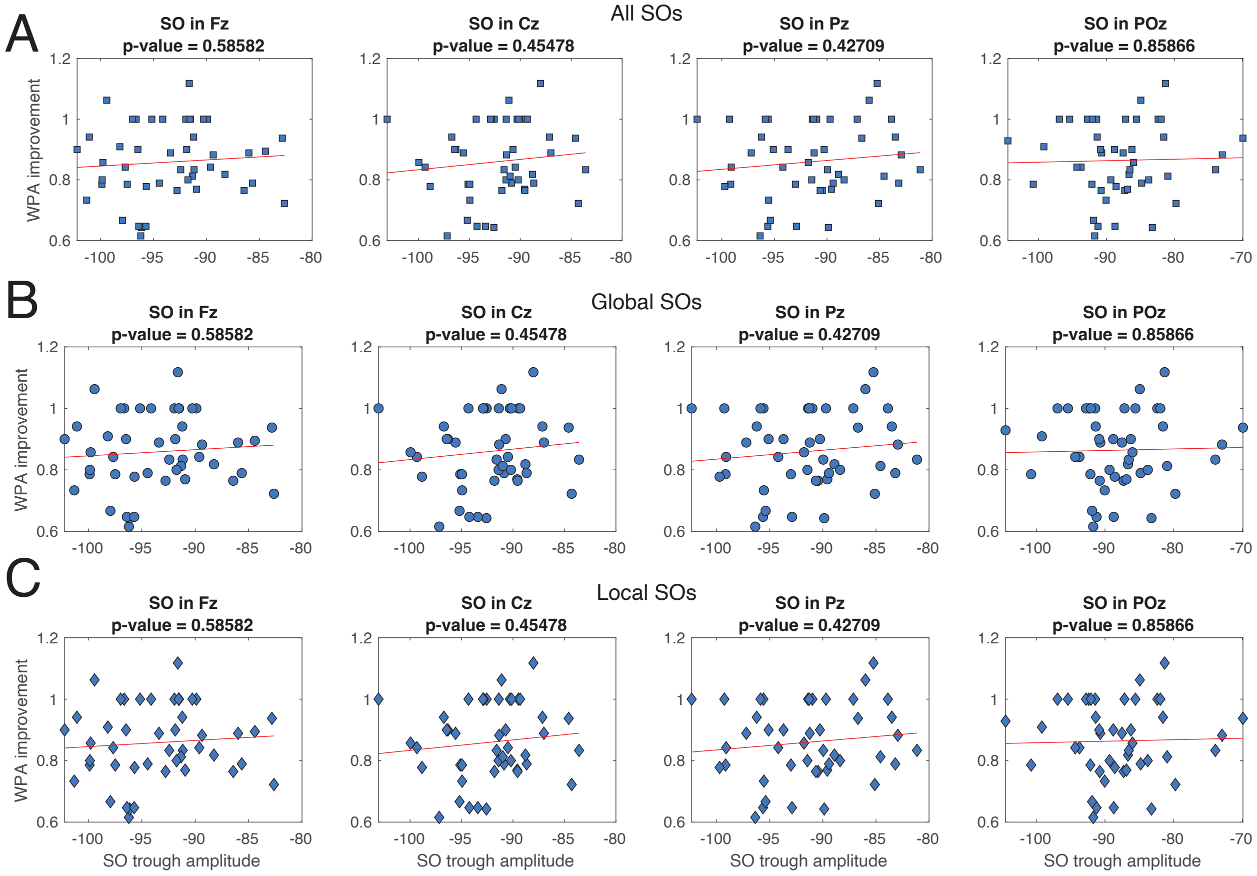

Figure S. 8 Correlations between SO amplitude and WPA improvement in each of the source/SO channels separately. A) Correlations between all SO amplitudes and WPA improvement (no significant effects). B) Correlations between Global SO amplitude and WPA improvement (no significant effects). C) Correlations between Local SO amplitude and WPA improvement (no significant effects).

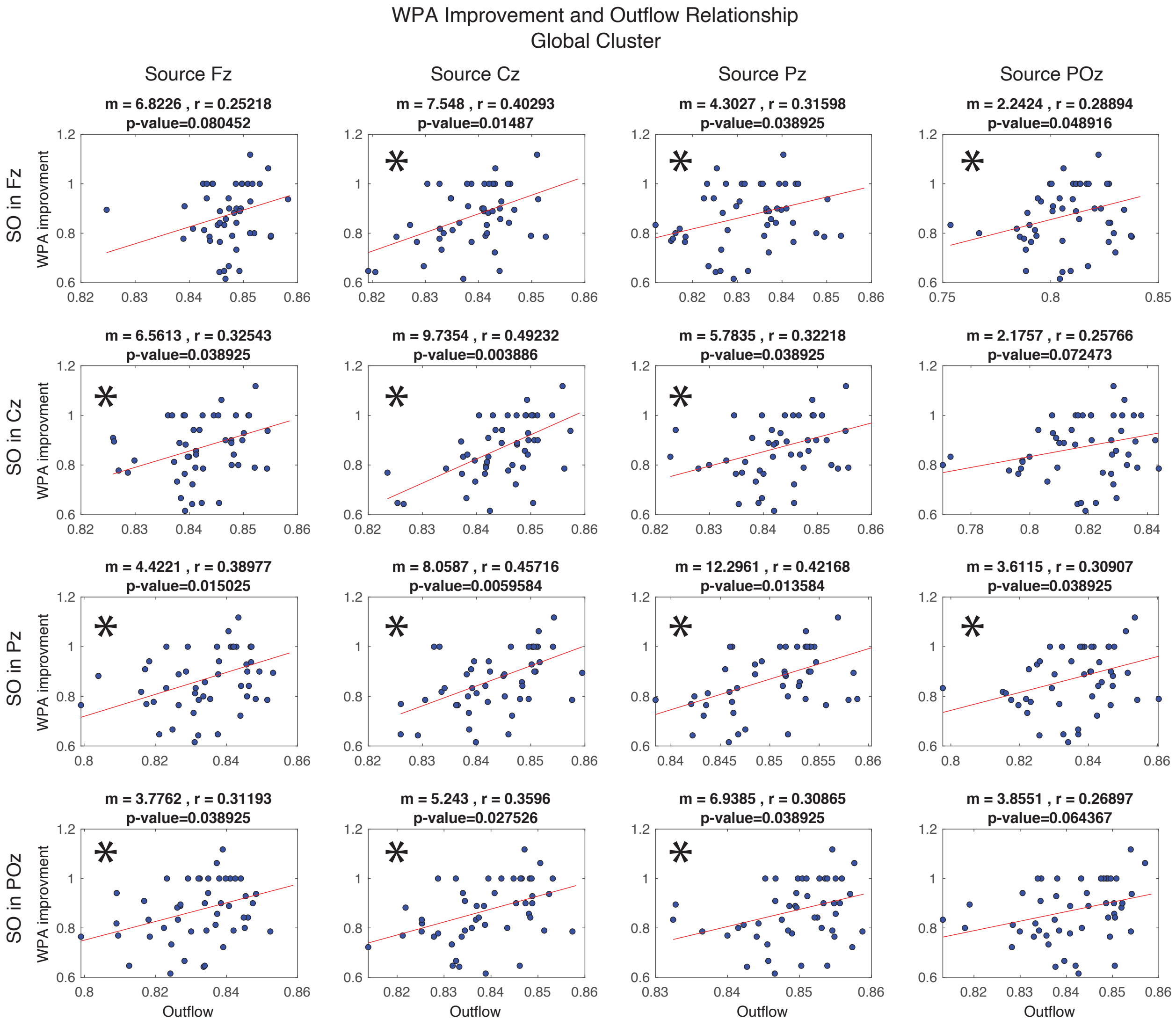

Figure S. 9) The results of the testing linear relationship between outflow from each source channel in different conditions of SO channel for the global SO cluster. Each row and column represents condition of SO and source channel respectively. The significant linear relationships are marked with asterisk (with p-values adjusted to FDR).

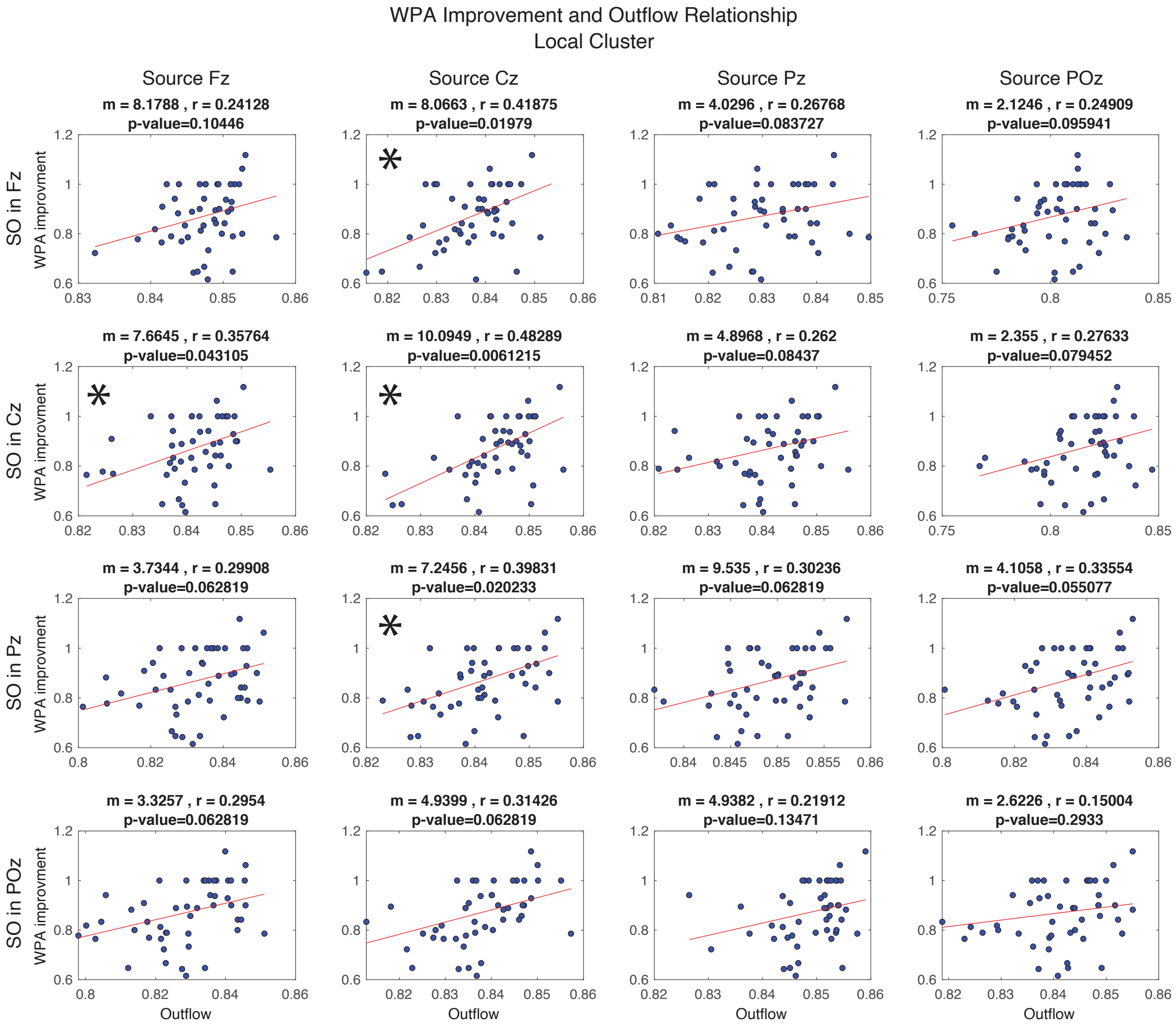

Figure S. 10 The results of the testing linear relationship between outflow from each source channel in different conditions of SO channel for the local SO cluster. Each row and column represents condition of SO and source channel respectively. The significant linear relationships are marked with asterisk (with p-values adjusted to FDR).

Table S. 1 Coefficients and p-values for linear predictors in LME models for amplitude of the outflow peaks.

	**Outflow peaks**	
**Linear predictor**	Coefficient	p-value
SO channel	0.0022	**<0.0001**
Source channel	-0.0019	0.0532
$D_{CH_{source},CH_{SO}}$	-0.0054	**<0.0001**
Peak phase (pre-/post-SO trough)	-0.0038	**<0.0001**

Table S. 2 Coefficients and p-values for linear predictors in LME models for peak information flow amount in SO phases $\pm{}^{\pi}/_{2}$. $D_{CH_{source},CH_{SO}}$ *is distance between source channel and SO channel,* $D_{R_{sink},CH_{SO}}$ *is distance between sink region and SO channel, and* $D_{CH_{source},CH_{R_{sink}}}$ *is distance between source channel and sink region.*

	Flow peaks	
**Linear predictor**	Coefficient	p-value
SO channel	0.0017	0.1457
Source channel	0.0002	0.2211
Sink region	0.0038	0.0543
$D_{CH_{source},CH_{SO}}$	-0.0006	**<0.0001**
$D_{R_{sink},CH_{SO}}$	0.0076	**<0.0001**
$D_{CH_{source},CH_{R_{sink}}}$	3.86e-05	0.8292
pre-/post-peaks	-0.0029	**<0.0001**

Table S. 3 Coefficients and p-values for linear predictors in LME models for amplitude of the flow peaks after adding the cluster as a fixed effect.

	Flow peaks	
**Linear predictor**	Coefficient	p-value
SO channel	0.0015	0.1421
Source channel	0.0001	0.2910
Sink region	0.0033	0.077
$D_{CH_{source},CH_{SO}}$	-0.0005	**<0.0001**
$D_{R_{sink},CH_{SO}}$	0.0067	**<0.0001**
$D_{CH_{source},CH_{R_{sink}}}$	1.65e-05	0.9042
Cluster	-0.0008	**<0.0001**
peak phase (pre- or post- SO trough)	-0.0026	**<0.0001**

Table S. 4 Coefficients and p-values for linear predictors in LME models for amplitude of the flow peaks after adding SO-spindle coupling as a fixed effect.

	Flow peaks	
**Linear predictor**	Coefficient	p-value
SO channel	0.0016	0. 0854
Source channel	0.0001	0. 2364
Sink region	0.0035	0.0686
$D_{CH_{source},CH_{SO}}$	-0.0005	**<0.0001**
$D_{R_{sink},CH_{SO}}$	0.0065	**<0.0001**
$D_{CH_{source},CH_{R_{sink}}}$	1.65e-05	0.7205
Cluster	-0.0008	**<0.0001**
peak phase (pre- or post- SO trough)	-0.0027	**<0.0001**
SO-spindle coupled	0.0008	**<0.0001**
